# Heterogeneity of magnitude, allergen immunodominance, and cytokine polarization of cockroach allergen‐specific T cell responses in allergic sensitized children

**DOI:** 10.1002/clt2.12073

**Published:** 2021-10-13

**Authors:** Ricardo da Silva Antunes, Aaron Sutherland, April Frazier, Veronique Schulten, Anna Pomés, Jill Glesner, Agustin Calatroni, Matthew C. Altman, Robert A. Wood, George T. O'Connor, Jacqueline A. Pongracic, Gurjit K. Khurana Hershey, Carolyn M. Kercsmar, Rebecca S. Gruchalla, Michelle Gill, Andrew H. Liu, Edward Zoratti, Meyer Kattan, Paula J. Busse, Leonard B. Bacharier, Stephen J. Teach, Lisa M. Wheatley, Alkis Togias, William W. Busse, Daniel J. Jackson, Alessandro Sette

**Affiliations:** ^1^ Division of Vaccine Discovery La Jolla Institute for Immunology La Jolla California USA; ^2^ Basic Research Indoor Biotechnologies, Inc. Charlottesville Virginia USA; ^3^ Rho Federal Systems Division Chapel Hill North Carolina USA; ^4^ Benaroya Research Institute Systems Immunology Division Department of Medicine University of Washington Seattle Washington USA; ^5^ Division of Pediatric Allergy, Immunology and Rheumatology Department of Pediatrics Johns Hopkins University School of Medicine Baltimore Maryland USA; ^6^ Boston University School of Medicine Pulmonary Center Boston Massachusetts USA; ^7^ Advanced General Pediatrics and Primary Care Ann & Robert H. Lurie Children's Hospital of Chicago Chicago Illinois USA; ^8^ Division of Asthma Research Cincinnati Children's Hospital Cincinnati Ohio USA; ^9^ Division of Pulmonary Medicine Cincinnati Children's Hospital Cincinnati Ohio USA; ^10^ Divisions of Infectious Diseases and Pulmonary Vascular Biology Department of Pediatrics University of Texas Southwestern Medical Center Dallas Texas USA; ^11^ Department of Pediatrics Children's Hospital Colorado University of Colorado School of Medicine Aurora Colorado USA; ^12^ Henry Ford Health System and Wayne State University School of Medicine Detroit Michigan USA; ^13^ College of Physicians and Surgeons Columbia University New York New York USA; ^14^ Division of Clinical Immunology and Allergy Icahn School of Medicine at Mount Sinai New York New York USA; ^15^ Department of Pediatrics Monroe Carell Jr Children's Hospital at Vanderbilt University Medical Center Nashville Tennessee USA; ^16^ Center for Translational Research Children's National Hospital Washington DC USA; ^17^ Division of Allergy, Immunology, and Transplantation National Institute of Allergy and Infectious Diseases National Institutes of Health Rockville Maryland USA; ^18^ Departments of Pediatrics and Medicine University of Wisconsin School of Medicine and Public Health Madison Wisconsin USA; ^19^ Department of Medicine University of California San Diego La Jolla California USA

**Keywords:** allergens, asthma, clinical immunology, cockroach, T cell

## Abstract

**Background:**

Characterization of allergic responses to cockroach (CR), a common aeroallergen associated with asthma, has focused mainly on IgE reactivity, but little is known about T cell responses, particularly in children. We conducted a functional evaluation of CR allergen‐specific T cell reactivity in a cohort of CR allergic children with asthma.

**Methods:**

Peripheral blood mononuclear cells (PBMCs) were obtained from 71 children, with mild‐to‐moderate asthma who were enrolled in a CR immunotherapy (IT) clinical trial, prior to treatment initiation. PBMC were stimulated with peptide pools derived from 11 CR allergens, and CD4+ T cell responses assessed by intracellular cytokine staining.

**Results:**

Highly heterogeneous responses in T cell reactivity were observed among participants, both in terms of the magnitude of cytokine response and allergen immunodominance. Reactivity against Bla g 9 and Bla g 5 was most frequent. The phenotype of the T cell response was dominated by IL‐4 production and a Th2 polarized profile in 54.9% of participants, but IFNγ production and Th1 polarization was observed in 25.3% of the participants. The numbers of regulatory CD4+ T cells were also highly variable and the magnitude of effector responses and Th2 polarization were positively correlated with serum IgE levels specific to a clinical CR extract.

**Conclusions:**

Our results demonstrate that in children with mild‐to‐moderate asthma, CR‐specific T cell responses display a wide range of magnitude, allergen dominance, and polarization. These results will enable examination of whether any of the variables measured are affected by IT and/or are predictive of clinical outcomes.

## 1 INTRODUCTION

Cockroach is a common allergen in urban and under‐resourced areas and a significant source of atopic morbidity worldwide, particularly among children and young adults.^1^, ^2^, ^3^ The German cockroach (CR, *Blattella germanica*) is commonly associated with CR allergies in the United States with CR allergens being detected in 85% of homes in low‐income urban communities.^4^ CR allergy has a high prevalence, and has long been established as a strong cause of asthma initiation and progression with early exposure leading to increased CR sensitization, asthma severity, and morbidity.^4^, ^5^, ^6^, ^7^, ^8^

Several studies defined CR allergens based on IgE reactivity from sensitized individuals and correlated sensitization prevalence with severity of clinical symptoms.^3^, ^9^, ^10^, ^11^, ^12^ T cells, and in particular type 2T helper cells, significantly contribute to the development of allergy and asthma^13^, ^14^, ^15^; however, CR‐specific T cell responses have been characterized in less detail,^16^, ^17^, ^18^, ^19^, ^20^, ^21^ and very little information is available particularly for the population most impacted by CR allergies, namely urban and under‐resourced children.

In general, allergen‐specific T cell responses are often characterized following in vitro expansion steps^17^, ^22^, ^23^, ^24^, ^25^ to account for their low frequency which may also alter the phenotype of responding T cells.^26^ We and others previously demonstrated that allergen‐specific T cells can be detected ex vivo using a novel assay strategy with the combination of several T cell epitopes into pools.^24^, ^27^, ^28^, ^29^ This technique uses the upregulation of the activation markers such as CD154 (CD40L) as a read‐out for T cell reactivity and can be combined with intracellular cytokine staining (ICS) to further identify T cell phenotypes as well as polyfunctionality^24^, ^27^, ^30^ and to improve the characterization of CR‐specific T cell responses.

Thirteen groups of German CR allergens have been defined based on IgE reactivity and are listed at the official allergen database maintained by the World Health Organization and International Union of Immunological Societies (WHO/IUIS) Allergen Nomenclature Sub‐committee (www.allergen.org).^2^ As in the case of other allergies (i.e., cat, mite, or mouse^24^, ^27^, ^31^, ^32^), certain allergens have been described as dominant for CR‐specific T cell responses. Those studies however were associated with certain limitations, such as testing a set of candidates derived from predicted epitopes from a more limited set of allergens, reliance on in vitro expansion and re‐stimulation steps, and most importantly they only addressed dominance in sensitized adults.^16^, ^17^, ^18^

Here we characterized the patterns of T cell responses to 11 cockroach allergens in a cohort of children with CR sensitization and asthma with a median age of 12 years that were enrolled as potential participants in an IT clinical trial (CRITICAL) and before initiation of treatment. T cell responses for each individual allergen were assessed by conducting ex vivo assays directly from PBMC not pre‐stimulated with CR extract using pools of overlapping peptides spanning the entire protein sequence of the various CR allergens. The basal numbers of regulatory CD4+ T cells (Tregs) was also assessed, and compared with the magnitude of effector T cell responses. Additional correlation analyses were performed to examine the relationship of T cell responses to skin prick test and serological IgE responses^33^ as well as to the Composite Asthma Severity Index (CASI) and its components.^34^

## 2 METHODS

### 2.1 Study subjects

This cohort is part of an ongoing clinical trial CRITICAL (Cockroach Immunotherapy in Children and Adolescents; https://clinicaltrials.gov/ct2/show/NCT03541187). Subjects were enrolled if they were IgE sensitized to CR antigen (measured by skin prick testing and serum IgE) and were ages 8 through 17 with well‐controlled asthma (mild‐moderate persistent) at study entry (Table 1). Clinical characteristics and evaluation are detailed in the online supporting information. The study was approved via a central Institutional Review Board, the Western IRB (WIRB Tracking Number 20180698), the DAIT NIAID and NIH/IND# 17979 (Protocol ID#: ICAC‐28). All participants enrolled in this study provided written consent or provided assent with parental consent.

**TABLE 1:** Characteristics of the donor cohort

Participating donors	Sex (%)	Demographic subgroup (%)	Other variables	Age	CASI score^a^	Cockroach‐specific IgE (kU_A_/L)	SPT wheal size^b^ (mm)
Total number *n* = 71	54.9% M 45.1% F	Black or African American (63.4) White (18.3) Other (18.3)	Range	8–17	2–8	0.12–>100	3.0–17.5
			Median	12.0	4.0	2.8	6.0
			Average	12.0	3.7	23.5	6.9
			Std deviation	2.4	1.3	69.6	3.3

^a^ CASI stands for Composite Asthma Severity Index. Range of scores: 0 (least severe) to 20 (most severe) asthma.

^b^ SPT stands for skin prick test.

### 2.2 PBMC isolation

Peripheral blood mononuclear cells (PBMC) were isolated from whole blood by using CPT (cell preparation tubes) tubes according to the manufacturer's instructions (BD Vacutainer CPT tube with sodium heparin BD 362753, BD Biosciences) as detailed in the online supporting information.

### 2.3 Measurement of IgE, IgG, and IgG4 antibody and peptide synthesis

Cockroach‐specific IgE, IgG, and IgG4 antibody levels were measured in sera using a Thermo Fisher Scientific ImmunoCAP system (Phadia 250 Immunoassay Analyzer; Thermo Fisher Scientific) as explained in the online supporting information. Sequences of 11 major cockroach allergens were collected from UniProt. A strategy using 15mer peptides overlapping by 10 amino acids was selected to get the full coverage of all the allergens (Table S1). It is well established that epitopes bind to HLA‐class II molecules through a nine residues core, and are on average 14–16 residues in overall length. The peptide selection utilized in this manuscript and commonly utilized in other studies relies on the fact that ensures that each 9 “core” amino acids that bind to the MHC‐II groove is contained in at least one peptide. An alternative strategy would be to generate 15mers overlapping by 14 amino acids. Although more rigorous, this strategy would significantly increase the number of peptides to be generated and tested which was not feasible in light of the limited numbers of cells available to study. Peptides were purchased from A & A as crude material on a small (1 mg) scale.

### 2.4 Activation Induced Marker (AIM) assay and experimental design

Evaluations of T cell responses were based on previously described Activation Induced Marker (AIM) ex vivo assays,^30^, ^35^, ^36^ utilizing the CD154 (CD40L) and CD137 (4‐1BB) markers, combined with intracellular cytokine staining (ICS), using the antibodies described in Table S2 and performed as detailed in the online supporting information. The cytokines IL‐4, IFNγ, and IL‐10 were included as representative of Th2, Th1, and Tr1/T Regulatory (Tregs) CD4 helper T cell subsets, respectively.^24^, ^37^ The phenotypic CD127 and CD25 Tregs markers were also included in the cytometry analysis panel^30^, ^38^ and the several T cell parameters determined as described in the supporting information.

### 2.5 Statistical analysis

Comparisons between cytokine responses were performed using the nonparametric two‐tailed, paired Wilcoxon test. Correlations between magnitude of response and polarization or between different cytokine production in each participant were performed using the Spearman's rank correlation coefficient test. No adjustment for multiple comparisons was performed. Pearson correlation test was used to compare the two different measurements of Tregs. Prism 8.0.1 (GraphPad) was used for these calculations. Values pertaining to significance and correlation coefficient (*R*) are noted in the respective figure, and *P* < 0.05 defined as statistically significant.

## 3 RESULTS

### 3.1 Study strategy

**FIGURE 1:**
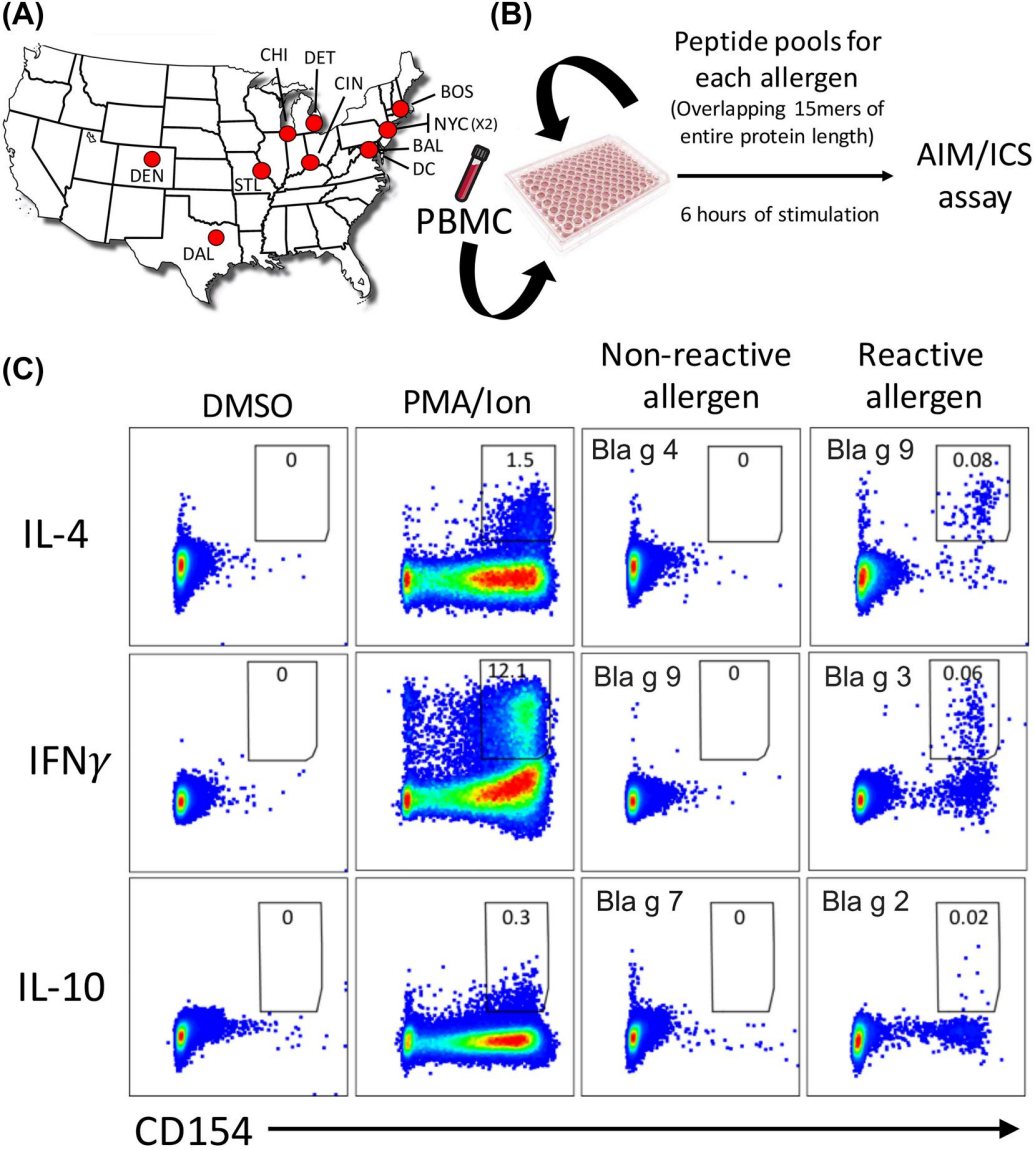
Study design and experimental strategy. (A) A total of *n* = 71 subjects were enrolled from 11 clinical sites, and blood samples were collected and PBMC isolated and cryopreserved. (B) PBMC were tested against a set of peptide pools consisting of overlapping peptides spanning the entire sequence of 11 different CR allergens, and T cell cytokine responses assessed by a combination of the Activation Induced Marker (AIM) and Intracellular Cytokine Staining (ICS) assays after 6 h of stimulation. (C) Dot plots depict the cytokine detection of IL‐4, IFNγ, and IL‐10 in CD154+ CD4+ T cells after stimulation with DMSO, PMA/Ion or sets of peptide pools for a given allergen. Three different donors are shown to illustrate the measurements of IL‐4, IFNγ, and IL‐10 cytokine secretion to a particular high‐reactive CR allergen and the absence of response to a particular CR allergen as control. Specific allergen tested in each condition is indicated in dot‐plots as well as the frequency of CD154+ cytokine + events of total CD4+ T cells

PBMC were obtained from 71 study participants enrolled from 11 clinical sites prior to allergen IT and cryopreserved for subsequent analysis (Figure 1a). To characterize the magnitude and polarization of allergen responses directed against *Blatella germanica* (Bla g) allergens, we tested sets of overlapping peptides, spanning the entire sequence of 11 different CR allergens, using Activation Induced Marker (AIM) assays,^30^, ^35^, ^36^ combined with Intracellular Cytokine Staining (ICS; Figure 1b and see supporting information for more detail).

Figure 1c shows representative data for the detection of each cytokine. Three different donors where used for the different cytokine measurements. Specifically, IL‐4 response to Bla g 9, IFNγ response to Bla g 3, and IL‐10 response to Bla g 2 allergen peptide pool stimulation. Strong cytokine production was observed in response to the positive control (PMA/Ion) but not in response to the negative control (DMSO) or peptide pools from allergens not recognized for a particular donor. For each participant, individual IL‐4, IFNγ, and IL‐10 cytokine responses to each pool was summated to determine the total number of effector T cells (Teff; antigen‐specific CD154+ cells^24^, ^30^).

### 3.2 Overall magnitude and polarization of Bla g‐specific T cell responses

**FIGURE 2:**
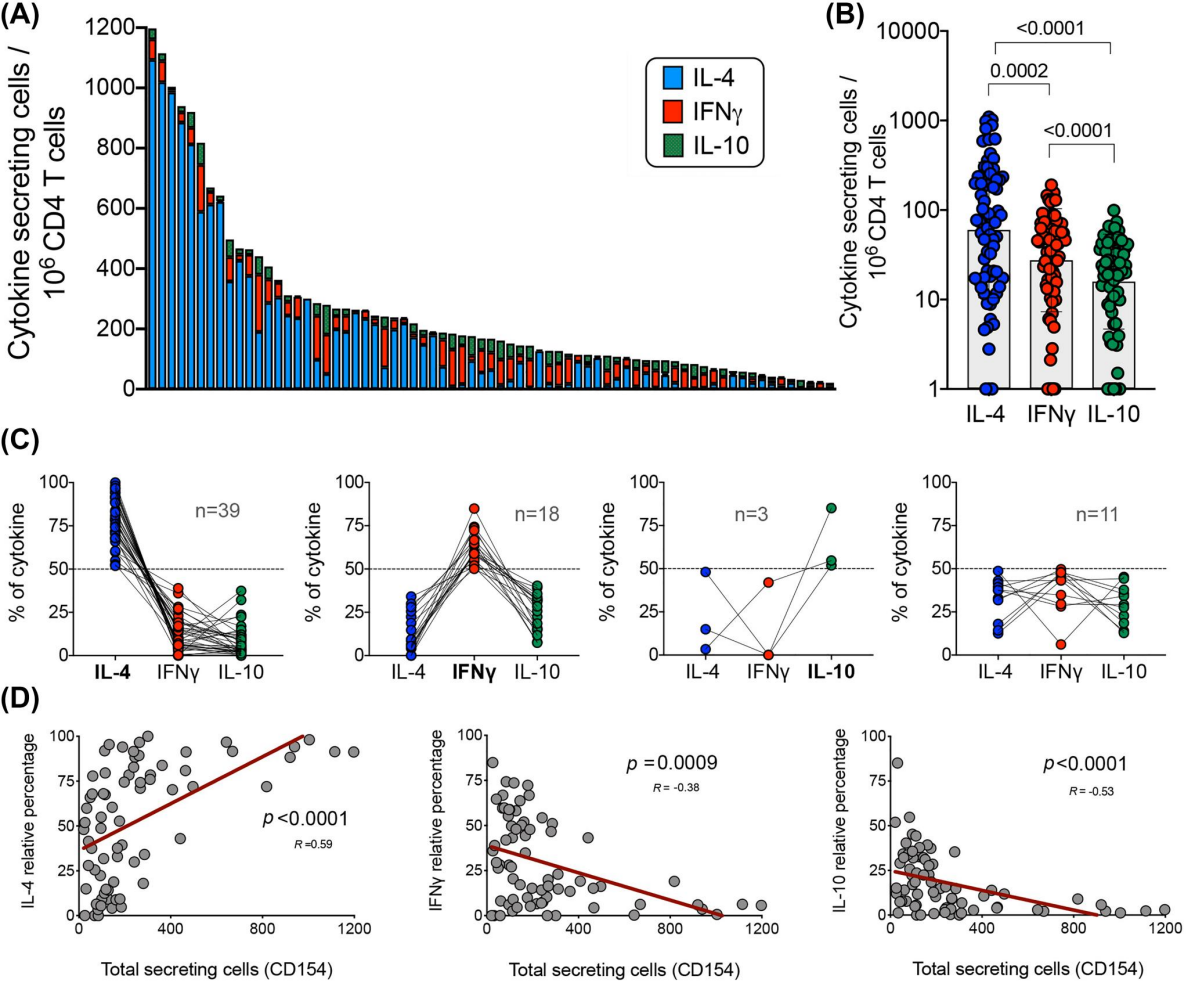
Baseline cytokine responses of CD154+ cells are IL‐4 polarized and correlate with magnitude of responses. (A) Graph bars show the overall magnitude of CD4+ T cell CR‐specific responses as the sum of the 11 individual allergens tested. Each bar represents a participant (*n* = 71) and the fraction of the response accounted for each cytokine (IL‐4 (blue), IFNγ (red), or IL‐10 (green)). (B) Graph compares the IL‐4, IFNγ and IL‐10 allergen responses for all the individual participants combined. Geometric mean and *p* value are shown as statistically significant by Wilcoxon's paired *t*‐test. (C) Graphs depict the percentage of each individual cytokine contribution from total response, grouped as function of the IL‐4, IFNγ, IL‐10 dominant (>50% of total response) or non‐dominant cytokine profile for a particular participant. Each dot and interconnected lines represents a participant and the number of participants in each group is shown. (D) Graphs show the correlation of total cytokine response with the relative IL‐4, IFNγ and IL‐10 cytokine response. *R* values and *p* values are shown as statistically significant by nonparametric Spearman correlation test and best fit represented by a linear regression line (red)

The overall magnitude of CD4+ T cell CR‐specific responses is shown in Figure 2a, where for each participant a bar represents the total cytokine response for all the different allergens combined. Each bar is further color coded to show which fraction of the response for each participant is accounted for IL‐4 (blue), IFNγ (red), or IL‐10 (green). The overall magnitude of baseline responses varied over approximately 2 logs across different subjects in the study cohort (ranging from 21 to 1198 cells per million of CD4+ T cells).

This large variation between participants was not the result of assay‐to‐assay variability, as demonstrated by specific controls utilized in the assays. More specifically, in each assay, we also included a PBMC aliquot from an adult volunteer with a moderate degree of cockroach allergy, who underwent apheresis to provide numerous cell aliquots available for use. As shown in Figure S1a, limited assay‐to‐assay variability was observed in repeated assays (*n* = 42) from the lowest to the highest response in the 3.3‐fold range, which is far less variable than variability observed within the participant cohort (57‐fold). Furthermore, as shown in Figure S1b, the variation in the percent of each cytokine for the control donor was calculated for each repeated assay. The response accounted predominantly for IL‐4 cytokine secretion and, the Th2 polarization (>50% of total response) was consistently observed in all technical repeats (*n* = 42). Lastly, no significant difference was observed in the magnitude of response as a function of the different clinical sites from which the subjects were enrolled (Figure S1c).

Overall, IL‐4 responses across the different participants were significantly larger than the IFNγ and IL‐10 responses, approximately 2‐fold and 4‐fold larger, respectively (Figure 2b). This was expected as all subjects in the study cohort were diagnosed with allergic asthma, and that a Th2 profile is a characteristic feature of allergic responses.^39^, ^40^ To visualize the nature and degree of polarization as a function of each individual participant, the same data were plotted in an alternative format (Figure 2c) where, for each participant, the percent of the total response to be ascribed to each of the three cytokines was plotted and grouped according with their polarization profile (>50% of total response). The responses in most participants (*n* = 39/71) were IL‐4 polarized (i.e., IL‐4 is the dominant cytokine produced), while only 18 and 3 participants were IFNγ and IL‐10 polarized, respectively. No clear polarization was noted for an additional 11 participants. Interestingly, an IFNγ and IL‐4 co‐dominant pattern was observed in 4 out of those 11 individuals.

The data in Figure 2a indicate that the most vigorous responses were also the most polarized toward IL‐4 production. Further analysis (Figure 2d) found that the total magnitude (total cytokine response) correlated with Th2 (IL‐4) responses (*p* < 0.0001; *R* = 0.59) and inversely correlated with Th1 (IFNγ) and IL‐10 producing cells (*p* = 0.0009; *R* = −0.38 and *p* < 0.0001; *R* = −0.53, respectively). These results suggest that stronger responses to CR allergens were more IL‐4 polarized, and that weaker responses were associated with IFNγ and/or IL‐10 production. Correlation between the different cytokine responses in each individual participant was also analyzed. As shown in 2, IFNγ and IL‐10 responses were highly correlated but IL‐4 did not correlate with IFNγ or IL‐10 responses. Further correlation analysis comparing the polyclonal T cell responses (PMA/Ion stimulation) and CR‐specific T cell responses was performed. As shown in Figure S3, CR‐specific immune responses in this study do not reflect the general immune responses at the individual donor level.

Lastly, in these experiments, we also included a previously described megapool of *Bordetella pertussis* (BP) epitopes.^41^ Children in our cohort, based on their year of birth, are expected to have been vaccinated with the acellular pertussis (aP) vaccine which is associated with a predominantly Th2 response.^42^, ^43^ As expected, and shown in Figure S4a, BP responses in this cohort were Th2 polarized, with a median ratio of IL‐4/IFNγ cytokine response of 2.5 and significant participant‐to‐participant variability. The degree of Th2 polarization observed in the BP responses did not correlate with the degree of polarization observed in the CR responses (Figure S4b), suggesting that there are factors determining the degree of Th2 polarization that are antigen/allergen‐specific.

### 3.3 Bla g 9 and Bla g 5 are the most dominant allergens for T cell responses

Previous studies established that certain allergens are immunodominant (i.e., account for a larger fraction of the total response)^17^, ^24^, ^44^ at the population level. However, more granular analyses also established that individual patterns of immunodominance are also detected, and the immunodominant antigens can vary from one individual to the next.^10^, ^11^, ^16^

**FIGURE 3:**
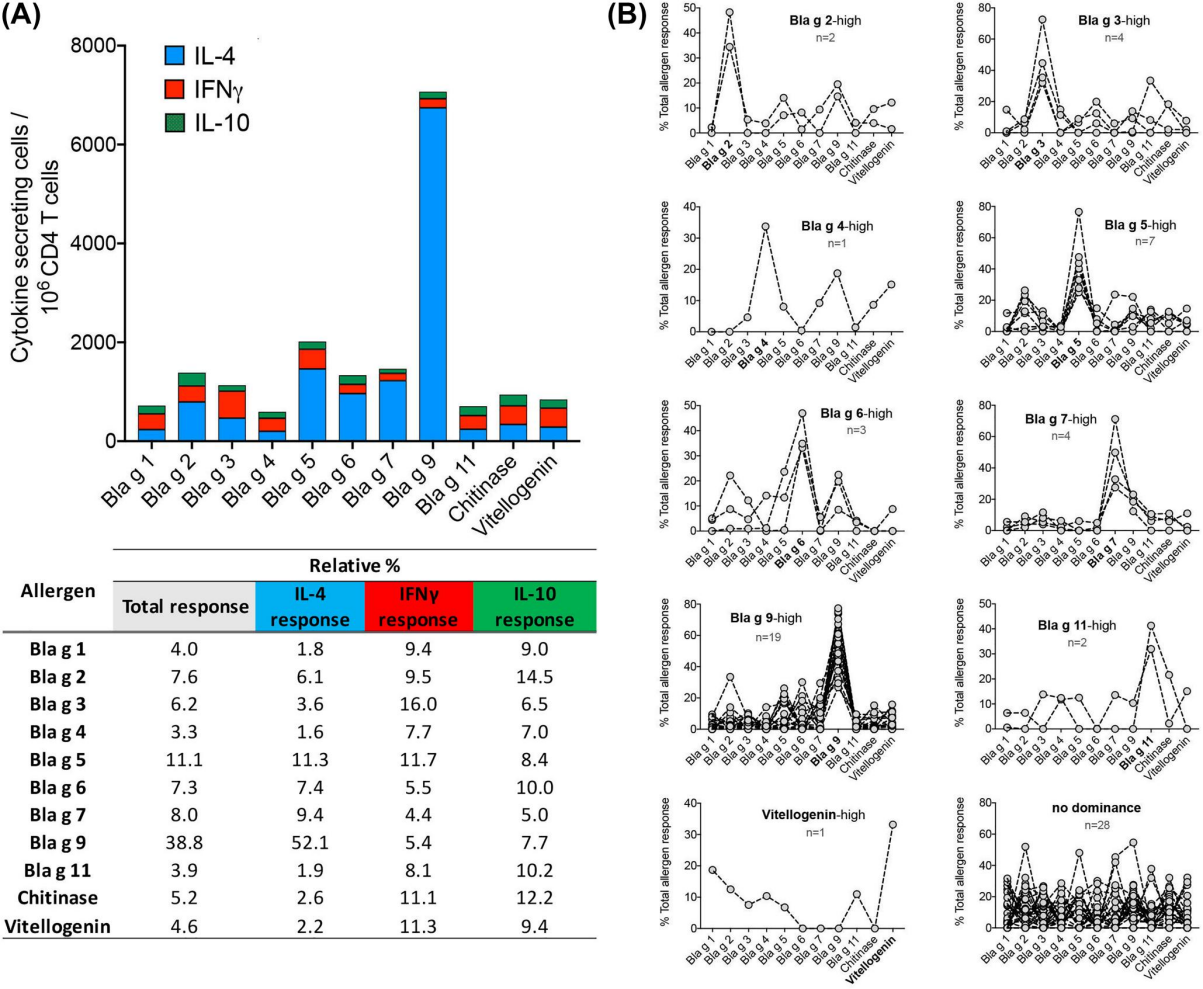
Bla g 9 and Bla g 5 are the most immunodominant allergens and dominance is participant specific. (A) Graph shows the overall response directed against the 11 allergens tested, calculated by summing each individual allergen response for all participants. Each bar represents an allergen and the fraction of the response accounted for each cytokine (IL‐4 [blue], IFNγ [red], or IL‐10 [green]). Table depicts the percentage of total or individual cytokine responses for each allergen. (B) Graphs show for each individual participant (dots and interconnected lines) the fraction of the response accounted for each allergen and grouped as a function of allergen immunodominance (‐high) or lack of dominance. Bla g 1 and Chitinase not shown in graphs as no dominant responses were observed. The number of participants associated with each category is shown

Accordingly, we first established which specific CR allergens would be dominant for T cell responses in the cohort of allergic CR‐sensitized children. Figure 3a shows the overall response directed against each allergen, calculated by summing, separately for each individual allergen, the responses observed in each individual participant. The Bla g 9 and Bla g 5 allergens were the most dominant, accounting for 38.8% and 11.1% of the total response. Other allergens were also recognized, albeit to a lower extent. When considering the individual types of immune responses Bla g 9 was by large the most dominant allergen of IL‐4 responses (52.1%), and Bla g 3 (16.0%) or Bla g 2 (14.5%) the most dominant allergens of IFNγ and IL‐10 cytokine responses, respectively (Figure 3a and Figure S5).

Next, the data were analyzed at a higher level of granularity to investigate which antigens would be more dominantly recognized for each of the subjects tested, and each participant was assigned to different immunodominance categories. In terms of total cytokine response (Figure 3b), a total of 19 participants recognized the Bla g 9 allergen as most dominant, while Bla g 5 was most dominant in 7 participants. Bla g 3 and Bla g 7 were dominant in 4 participants, and Bla g six in 3 participants. The other allergens were dominant in one and two participants each or none, and patterns of co‐dominance or no clear pattern of dominance were detected for 28 participants (Figure 3b).

### 3.4 Detection of basal levels of regulatory T cells

**FIGURE 4:**
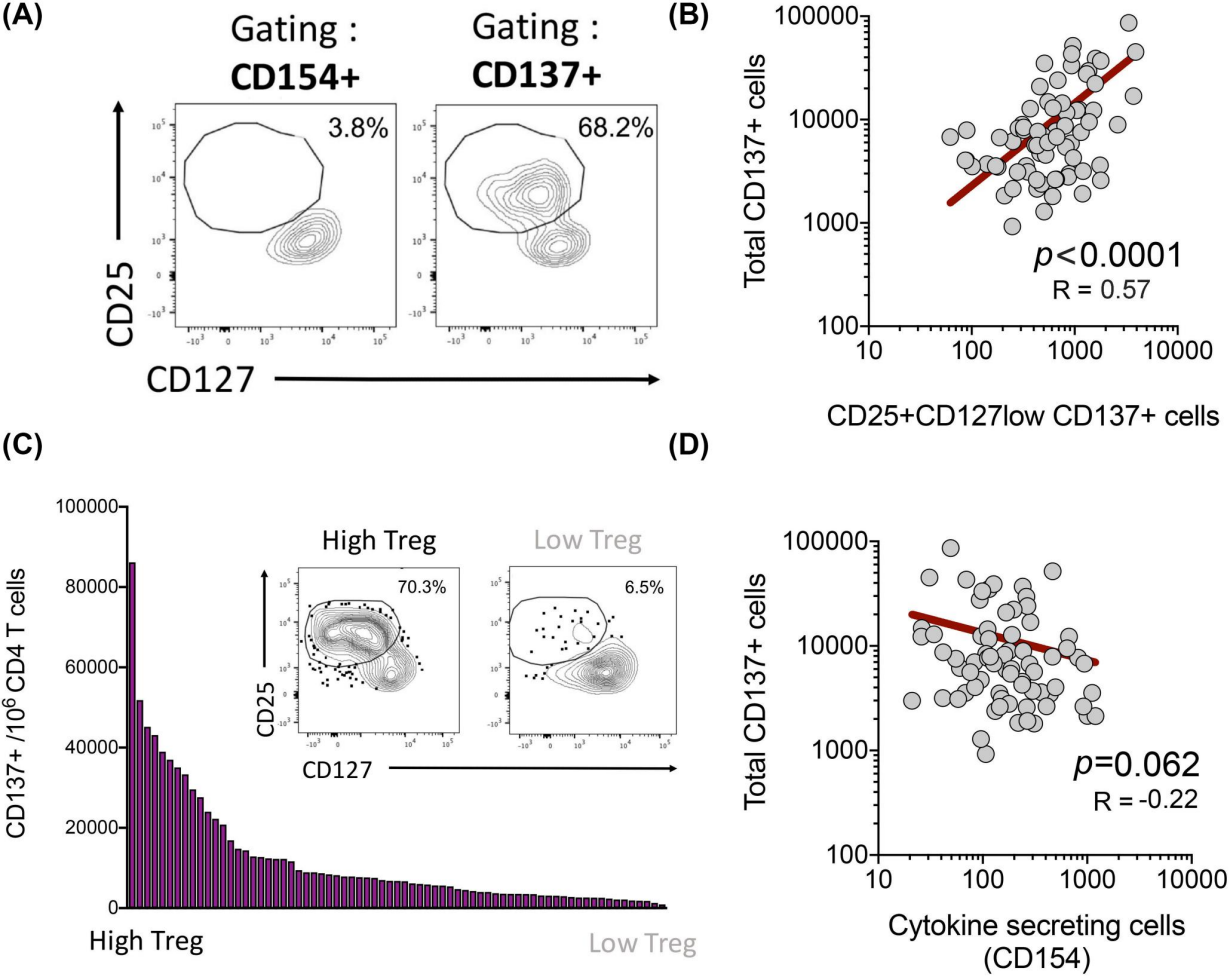
Treg numbers varies amongst participants and are inversely correlated with Teff responses. (A) Dot plots show a representative participant for the Treg gating strategy. (B) Graph show the correlation of total CD137+ cells and CD25 + CD127low CD137+ cells in unstimulated cells by Pearson correlation test. (C) Graph bars show the number of Tregs (CD137+ cells) across all participants (*n* = 71). Figure insert depicts 2 representative participants with High or Low Treg populations, respectively. (D) Graph shows the correlation of Tregs (Total CD137+ unstimulated cells) and Teff (cytokine + CD154+ allergen‐stimulated) cells by nonparametric Spearman correlation test. For both correlation graphics, each dot represents a participant (*n* = 71). *R* values and *p* values are shown and best fit represented by a linear regression line (red)

In the next series of experiments, we analyzed the number of Tregs at baseline in the absence of antigen stimulation. We defined baseline Tregs as the total number of CD137 + CD154‐ T cells detected at baseline, without any antigen stimulation.^30^ As expected, Tregs defined as CD137+ were correlated with Tregs defined by the alternative markers CD4+CD25 + CD127low^38^, ^45^ (Figure 4a and 4b). There was substantial variation in the number of Tregs (total CD137+) among participants (Figure 4c), the number of CD137+ cells varing 90‐fold from 925 to 86,000 per million CD4+ T cells.

We hypothesized that the size of the baseline Treg population present in each individual might influence their capacity to mount an allergen‐specific T cell response following stimulation with the allergen‐derived peptide pools. There was a suggestion of an inverse correlation between the number of Tregs (CD137+ unstimulated cells) and the number of Teff (cytokine + CD154+ allergen‐stimulated cells), but this was not statistically significant (Figure 4d). We further observed to be the case in patients with IL‐4 but not IFNγ dominant responses (Figure S6).

### 3.5 Correlation of T cell responses with sera antibody titers to CR extract, SPT responses, and clinical presentation

We also tested whether T cell responses were associated with IgE, IgG, and IgG4 titers specific for CR as well as with the ratio between IgE/IgG4 responses. The results summarizing these analyses are shown in Table 2. A significant correlation was observed between IL‐4 responses or IL‐4 (Th2) polarization and IgE levels (*p* = 0.0003; *R* = 0.42 and *p* < 0.0001; *R* = 0.45, respectively) and to a less extent with total IgG but not IgG4. Conversely, IFNγ (Th1) and IL‐10 polarization showed an inverse correlation with the IgE titers (*p* = 0.0044; *R* = −0.33 and *p* = 0.0013; *R* = −0.37, respectively). The same results were noted in regards to the correlation with the IgE/IgG4 ratios. In addition, as shown in Table 2, the total magnitude of responses positively correlated with IgE titers or the IgE/IgG4 ratios (*p* = 0.0082; *R* = 0.31 and *p* = 0.0071; *R* = 0.32, respectively), and no associations were observed for Treg frequencies.

**TABLE 2:** Correlation of T cell responses with cockroach (CR)‐extract specific titers

Spearman r	IgE Ext (RU^a^)	IgG Ext (RU^a^)	IgG4 Ext (RU^a^)	Ratio IgE/IgG4
	Total magnitude of responses (CD154)			
*R*	0.31	0.15	0.14	0.32
*P* value	0.0082	0.2125	0.2542	0.0071
	IL‐4 responses (CD154)			
*R*	0.42	0.2	0.12	0.43
*P* value	0.0003	0.0965	0.3141	0.0002
	IL‐4 (Th2) polarization			
*R*	0.45	0.26	0.12	0.43
*P* value	<0.0001	0.0301	0.3378	0.0002
	IFNγ responses (CD154)			
*R*	−0.1	−0.13	0.07	−0.1
*P* value	0.3952	0.2704	0.5647	0.398
	IFNγ (Th1) polarization			
*R*	−0.33	−0.26	−0.085	−0.33
*P* value	0.0044	0.0263	0.4784	0.0047
	IL‐10 responses (CD154)			
*R*	−0.18	13	−0.00099	−0.2
*P* value	0.1328	0.2979	0.9935	0.096
	IL‐10 polarization			
*R*	−0.37	−0.18	−0.099	−0.41
*P* value	0.0013	0.1347	0.411	0.0003
	Treg baseline numbers			
*R*	−0.11	−0.15	−0.062	−0.093
*P* value	0.3729	0.2209	0.6047	0.4384

^a^ RU stands for relative units.

We next examined the correlation of T cell responses with the results of a skin prick test (SPT) specific for extracts of German and American CR, which share homologous allergens (www.allergen.org). As shown in Table S3, total responses or IL‐4 responses were positively but weakly correlated (*p* = 0.035; *R* = 0.25 and *p* = 0.042; *R* = 0.35, respectively), while IL‐10 polarization was negatively correlated (*p* = 0.015; *R* = −0.29) with the mean wheal diameter (MWD) of the SPT for the American CR extract. Conversely, none of the T cell variables correlated with MWD of the SPT for the German CR extract (Table S3). Despite differences in the source of the CR extract considered, these results confirm the associations noted at the serological level.

Lastly, we examined correlations of the T cell variables (magnitude, polarization, or dominance of specific allergens) with clinical variables such as those captured by Composite Asthma Severity Index (CASI) score and components.^34^ As shown in Table S4, none of the T cell immunological variables correlate with the total CASI score. Also, no differences were observed in clinical symptoms between patients with IL‐4 or IFNγ dominant responses (Figure S7). These results were not unexpected given the fact that this cohort was designed to encompass relatively homogeneous CASI scores, ranging from low to medium severity (median CASI of 4).

## 4 DISCUSSION

Here we report the direct ex vivo characterization of T cell responses to a panel of 11 previously described CR allergens in a cohort of CR allergic children and adolescents with well‐controlled asthma. In particular, we found over 57‐fold variation in magnitude of allergen‐specific T cell responses. The more vigorous responses were also the most Th2 polarized and while the allergens Bla g 9 and Bla g 5 were most immunodominant, individual subjects exhibited distinctive patterns of allergen dominance. Subjects with higher magnitude of allergen‐specific T cell reactivity had lower number of Treg populations. Overall, this study unveils a surprisingly high level of heterogeneity in the pattern of Bla g‐specific T cell reactivity, both quantitatively and qualitatively. Since the cohort analyzed is enrolled in a future IT clinical trial, this finding lays the foundation for examining whether this heterogeneity may potentially correlate with IT outcomes.

This study is to the best of our knowledge, the first characterization of *ex vivo* CR‐specific CD4+ T cell responses in CR‐sensitized urban and under‐resourced children with asthma, and for the majority of the known CR allergens to date, which are also components commonly found in CR extracts used for IT.^2^, ^10^ Our results also differ from previous attempts of portrayal of CR‐specific responses,^17^, ^19^, ^20^ because of the high level of granularity and because the *ex vivo* approach allowed to characterize responding cells to a minimal gap from bona fide *in vivo* responses.

We identified a large dynamic range in terms of magnitude of CD4+ T cell responses, despite the narrow age range and similar clinical manifestations of disease among participants. Positive controls included in each assay excluded that this was due to inter‐assay variability. The assessment of the variation of PBMC from the same subjects taken at different time points would have also been of great interest but limited by the number of existing cells available to perform the tests. Nevertheless, this should be considered in future study designs. Likewise, the variability was not explained by differential responses as a function of the different clinical sites from which each subject was enrolled. A high spread in the magnitude of T cell responses has been previously described for other allergic responses, namely to cat dander, fungus, mite and grass or birch pollen^30^, ^46^, ^47^ using ex vivo methodologies similar to our study. It is possible that the ex vivo assay strategy utilized contributed to reveal this heterogeneity, as previous allergy studies in the case of CR^16^, ^18^, ^20^ or other allergens^24^, ^27^ utilized in vitro restimulation protocols which might obscure and blunt differences.

We also analyzed the functionality of CD4+ T cell responses, which was dominated by IL‐4 production and a Th2 polarization profile. Development of early allergic disease seems to be related to sustained Th2‐skewed immunity during childhood^48^ and previous studies have also demonstrated that levels of Th2 cytokines such as IL‐4, IL‐5, and IL‐13 are associated with pathogenesis of both allergy and asthma.^13^, ^14^, ^15^ In our study, Th2 polarization was the dominant phenotype but other patterns were also observed. The accuracy of this characterization was confirmed with a positive control included in each assay. In particular, while IL‐10 responses were rare, a profile of IFNγ production or Th1 polarization was observed in one‐fourth of the participants and associated with weaker T cell CR‐specific responses. Also, since IFNγ is thought to be secreted in higher amounts per cell than IL‐4, perhaps the actual net amount of cytokine production might not necessarily correlate with the number of cytokine secreting cells as determined herein, but additional studies would be needed to clarify this conjecture. Interestingly, resolution of allergic‐related immunopathologies is often also attributed to increase of IFNγ levels rather than reduction of Th2‐cytokine production,^49^ and IL‐10 is found to regulate CD4+ Th2 cells during allergic airway inflammation.^50^ Therefore, the close monitoring of both IL‐10 and IFNγ cytokines would be of interest in the context of IT and could provide new clues for the association of baseline patterns of polarization or dominant allergens and IT outcomes.

Bla g 9 (arginine kinase) and Bla g 5 (glutathione‐S‐transferase) were the most immunodominant allergens. Interestingly, these enzymes are well conserved among arthropods or insects and have been shown to cross‐react with human IgE antibodies^51^, ^52^ and are often considered pan‐allergens and important players in the clinical manifestation of allergic sensitization.^53^ Also, previous studies have reported Bla g 5 as a dominant T cell allergen^10^, ^16^, ^17^ but patterns of allergen dominance could be associated with different forms of allergic disease.^17^ These observations were made in adult cohorts, and this raises the possibility that Bla g 9 recognition could be a pattern predominantly associated with childhood CR allergy.

This pattern of immunodominance has been observed in subjects from the different clinical sites, irrespective of geographical location, further suggesting that Bla g 5 and Bla g 9 responses to CR are intrinsic to this particular age cohort. Changes in the IgE pattern of reactivity to individual Timothy grass and birch pollen allergens were observed over the course of 20 years for sensitized individuals.^54^ Future studies could examine whether changes in allergen immunodominance are apparent as a function of age and exposure history.

The pattern of reactivity as a function of the specific subject considered was also heterogeneous with different individuals recognizing either Bla g 9 or Bla g 5 as dominant, with dominant responses to other allergens or no pattern of dominance also observed. Individual immunodominance patterns were observed in adult cohorts^16^ and for IgE reactivity.^55^ Different HLA could influence allergen immunodominance. Associations between CR sensitization and HLA class II antigens have been suggested^56^, ^57^ and consistently shown in food allergies.^58^, ^59^

In addition, we measured basal levels of non‐antigen specific regulatory T cells and observed that T cells inversely correlated with the effector CD4+ T cell responses although it did not reach statistical significance. These results were not unexpected and were consistent with previous observations from Bacher et al.^30^. The fact that the population of Treg cells varies amongst participants is also of potential interest, as this will enable monitoring of Treg and addressing whether baseline Treg levels predicts response to IT. A possible outcome is that IT may induce or enhance regulatory T cell function, which can be further tested. Indeed, peanut oral immunotherapy has been shown to increase antigen‐induced Treg function.^60^

Overall, the current study reveals that in a pediatric cohort of CR‐sensitized subjects with mild‐to‐moderate asthma, subjects exhibit substantial heterogeneity in many key immunological parameters associated with CR‐specific T cell responses. We hypothesize that this large dynamic range of T cell reactivity may influence outcomes of allergen‐specific IT. These findings will also enable future research examining which of these parameters best predict the trajectory of evolution of allergic disease, and responsiveness to therapeutic intervention.

## CONFLICT OF INTEREST

All authors declare no conflict of interest.

## Supporting information

Supplementary Material 1

Supplementary Material 2
