# Supplementary material for: Heterogeneity of magnitude, allergen immunodominance, and cytokine polarization of cockroach allergen‐specific T cell responses in allergic sensitized children
Source: Clin Transl Allergy. 2021 Oct 13;11(8):e12073. doi: 10.1002/clt2.12073 (PMC8514843; doi:10.1002/clt2.12073)
Supplement: Supplementary file 1 — Supplementary Material 1 [file CLT2-11-e12073-s002.docx]

**SUPPORTING INFORMATION**

**SUPPLEMENTARY MATERIAL AND METHODS**

**Cohort characteristics, clinical evaluation and study approval**

The participants were enrolled and selected based on residence in urban and under-resourced neighborhoods from 11 sites in 10 major urban areas in the United States (Baltimore, Boston, Chicago, Dallas, Denver, Detroit, New York (2 sites), Washington, D.C., Cincinnati, and St. Louis). Enrolled participants participated in a screening visit which included a blood draw and spirometry in accordance with ATS/ERS standard. Information was collected on asthma symptoms, albuterol/levalbuterol use, recent exacerbations, and the current level of controller therapy via questionnaire. The questionnaire and lung function (Forced expiratory volume; FEV1) data were used to calculate the Composite Asthma Severity Index (CASI) score (range 0-20) for each participant ^34^. Because entry criteria required well-controlled asthma and cockroach hypersensitivity, the participants are relatively homogeneous in terms of having relatively low CASI scores and presence of cockroach sensitization. All immunologic parameters were collected prior to initiation of allergen IT. The study was approved via a central Institutional Review Board, the Western IRB (WIRB Tracking Number 20180698), the DAIT NIAID and NIH/IND# 17979 (Protocol ID#: ICAC-28). All participants enrolled in this study provided written consent or provided assent with parental consent.

**PBMC isolation**

Blood was collected from whole blood by using CPT tubes (approximately 8 mL for each CPT tube (BD Vacutainer CPT tube with sodium heparin BD 362753, BD Biosciences, Franklin Lakes, NJ, USA)), gently inverted, and immediately centrifuged. Peripheral blood mononuclear cells (PBMC) were collected by fine aspiration from under the plasma layer, suspended in fetal bovine serum (FBS) containing 10% (vol/vol) dimethyl sulfoxide (DMSO) and stored in each clinical facility at -80ºC immediately after processing and then shipped to the La Jolla Institute (LJI) and cryopreserved in liquid nitrogen until use. Quality controls (QC) were performed in each sample to ensure quality based on determination of cell viability by trypan blue upon thawing and assessment of cell number yield.

**Activation Induced Marker (AIM) assay**

Evaluations of T cell responses, were performed based on previously described Activation Induced Marker (AIM) *ex vivo* assays ^30,35,36^ using peptides for the 11 major cockroach allergens **Supplementary Table 1**). Individual peptides were resuspended in DMSO at a final concentration of 40 mg/ml. The peptides encompassing each individual CR allergen were pooled, lyophilized, and the resulting pool of peptides resuspended to a stock concentration of 1 mg/ml and then used at a concentration of 2 µg/ml in the AIM assay. Peripheral blood mononuclear cells (PBMC) were thawed and rested overnight, plated at 2 × 10^6^ cells per well in a 96-well plate. 12-18h later, cells were stimulated with sets of peptide pools for different allergens, phorbol myristate acetate (PMA) and Ionomycin (Ion) (positive control), or medium alone with DMSO (negative control) in the presence of 1 µg/ml CD40 (Miltenyi Biotec, Auburn, CA, USA). Cells were incubated for 6 h, adding Brefeldin A (1 µg/ml) for the last 3 h. After the incubation, cells were labeled with anti-CD154, -CD137, -CD25, -CD127, -CD4, -CD3, CD8, -CD14, -CD19, and live/dead fixable viability dye (Life Technologies, San Diego, CA, USA) (**Supplementary Table 2**). After staining and washing, cells were fixed and permeabilized for intracellular staining, which was performed with anti-CD154, -CD137, -IL-4, -IFNγ, and -IL-10 (BD Biosciences, Franklin Lakes, NJ, USA) (**Supplementary Table 2**), and probed by flow cytometry using a BD LSR II flow cytometer.

**Experimental design**

With this AIM/ICS methodology, we determined: 1) Individual cytokine response: IL-4, IFNγ or IL-10 production calculated by summing positive signal in CD154+ CD4+ T cells for all CR allergen pools of each participant and expressed as number of cells per 10^6^ of total CD4^+^ T cells; 2) Magnitude of response: Sum of total IL-4, IFNγ and IL-10 cytokine responses (Teff; Effector T cells); 3) Polarization of response: Percentage of the total cytokine response ascribed to each of the three cytokines; 4) Dominance of response: Percentage of the overall response ascribed to each of the eleven allergens for all the cohort combined or individually for each participant. 5) Treg numbers: Identified as CD4+CD25+CD127low expressing cells ^45^ or alternatively as CD137+CD154- ^30^ and expressed as number of cells per 10^6^ of total CD4^+^ T cells. Data were analyzed using FlowJo software (TreeStar, Ashland, OR, USA).

**Measurement of IgE, IgG, and IgG4 antibody levels**

Cockroach-specific IgE, IgG and IgG4 antibody levels were measured using in-house extract-loaded streptavidin ImmunoCAPs by a Phadia 250 (Thermo Fisher Scientific, Portage, MI). To prepare the in-house ImmunoCAPs, biotinylated cockroach extract was loaded and incubated on streptavidin ImmunoCAPS using a Phadia 100. ImmunoCAPs were transferred to a Phadia 250 where measurements of IgE, IgG and IgG4 were performed according to the manufacturer’s instructions. Sera were tested undiluted for IgE and 1:100 for IgG and IgG4. Sera with cockroach-specific IgE >100 KU_A_/L were re-tested at 1:5 dilution. The lower limits of quantification were 0.1 kU_A_/L for IgE, 2 mg/L for IgG and 0.07 mg/L for IgG4. Streptavidin ImmunoCAPs not loaded with cockroach extract were used to assess IgG non-specific binding. Because values of IgG ≥ 5 mg/L were detected in 6% of the samples tested (and IgG ≥ 2 mg/L in 35%), values of IgG bound to empty streptavidin ImmunoCAPs were subtracted from the IgG values obtained with in-house prepared ImmunoCAPs. The steps performed to prepare the in-house cockroach-specific ImmunoCAPs are described in the following section.

**Preparation and optimization of in-house CR-specific ImmunoCAPs for antibody measurements**

**1. Allergen extract biotinylation**

Optimization of the cockroach extract biotinylation was carried out by adding EZ-Link Sulfo-NHS-LC-Biotin (Thermo Scientific, Rockford, IL) at various folds of molar excess (20-40) to the extract as is or to extract that was previously dialyzed into PMSF or diluted with PBS. The biotinylated mix was incubated for 30 minutes and put over a pre-washed Zeba Desalt Spin Column (Thermo Scientific, Rockford, IL) 2 times and the concentration was determined either by APA (Cytoskeleton Inc., Denver, CO) or by the Pierce BCA Protein Assay (Thermo Scientific, Rockford, IL).

**2. Optimization of the amount of biotinylated extract loaded to the streptavidin ImmunoCAP**

To optimize the amount of the extract loaded per ImmunoCAP, streptavidin ImmunoCAPS (ThermoFisher Scientific, Portage, MI) were loaded and incubated on the Phadia 100, with the biotinylated extract at 5-8 concentrations within the range 0.1-20 µg/CAP. Two human sera previously tested by ImmunoCAP for their allergen specificity (using 3 µg of extract/CAP) were selected and tested for binding to the extract at these amounts. Optimal amounts of extract loaded to the ImmunoCAP were determined from the results.

**3. Stability study of allergen loaded into the ImmunoCAP.**

Biotinylated extract was loaded and incubated on streptavidin ImmunoCAPS at its optimized concentration. CAPS were then stored at 4°C until run with 3 allergen-specific sera on Day 3. Results showed that the loaded ImmunoCAPS were stable at 4°C for up to 3 days.

**SUPPLEMENTARY FIGURES AND TABLES**

**
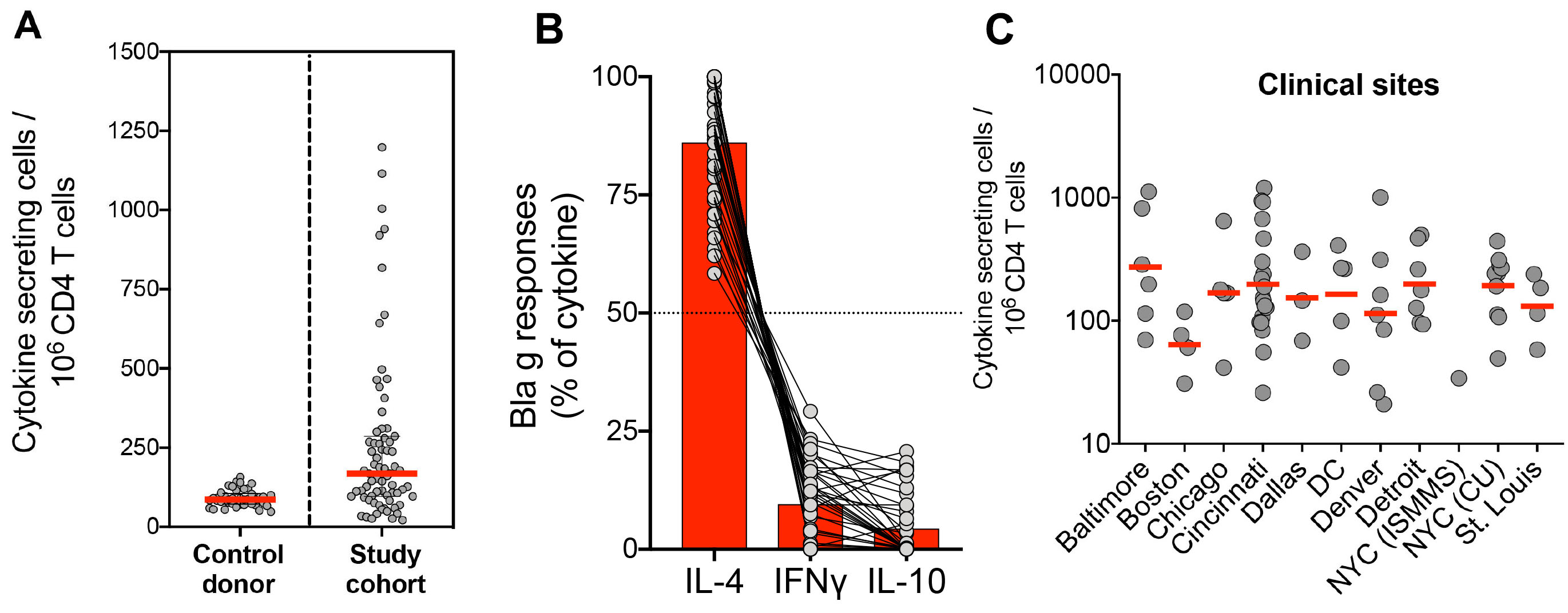
**

**Supplementary Figure 1.** **Control participant responses used to test assay to assay variability and segregation of responses by clinical site.** The PBMC aliquot of the control participant was stimulated with Bla g 2, Bla g 5, and Bla g 9 allergens, which were the most reactive CR allergens for this particular participant. A) Graph shows the repeated measurement (n=42) of CR responses for independent assays of a control participant included in the study (left) or the total CR responses for each individual participant (n=71) across the entire cohort (right). Average and Median is shown by a red line respectively. B) Graph shows the repeated measurement of cytokine responses of the control participant depicted as the percentage of each individual cytokine contribution from total response. Average is shown by a red bar and each dot and interconnected lines represents a repeated measurement of 42 independent experiments. C) Graph shows the total magnitude of response as a function of the different clinical sites from which the subjects were enrolled. Median is shown. Each dot represents a participant (n=71).

**
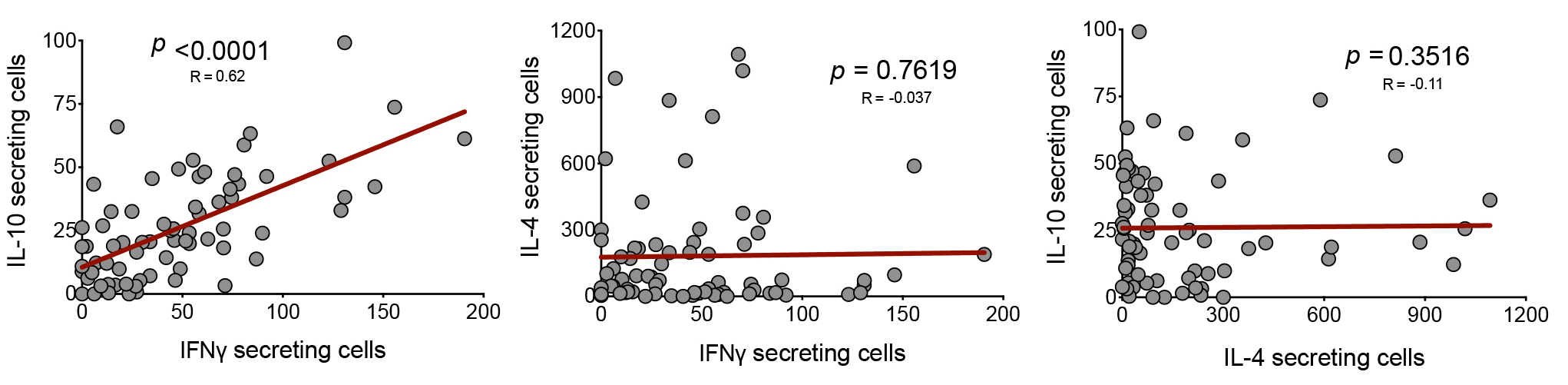
**

**Supplementary Figure 2. IFNγ**  **and IL-10 responses are highly correlated.** Graphs depict the correlation of the combination of two individual cytokine responses (IFNγ vs. IL-10, IFNγ vs. IL-4 and IL-4 vs. IL-10). Each dot represents a participant (n=71). R values and p values are shown as statistically significant by nonparametric Spearman correlation test. The best fit is represented by a linear regression line (red).

**Supplementary Figure 3. Polyclonal and CR-specific responses are not correlated.** Graphs depict the correlation of the total CR responses with the polyclonal responses (PMA/Ion) performed for each participant in the same assay as control. Each dot represents a participant (n=71). R values and p values are shown using the nonparametric Spearman correlation test. The best fit is represented by a linear regression line (red).

**
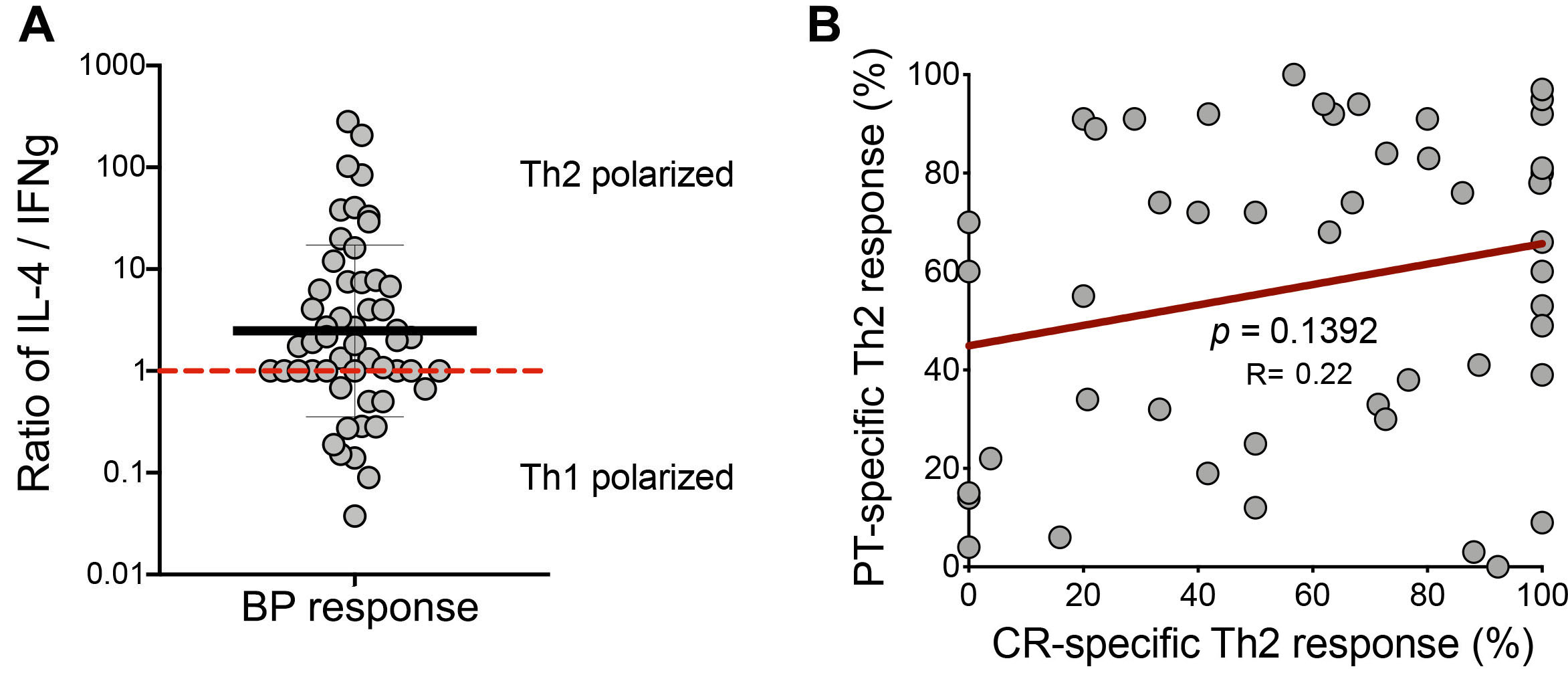
**

**Supplementary Figure 4. BP responses are Th2 polarized and do not correlate with CR Th2 polarization pattern.** A) Graph shows the ratio of IL-4/IFNγ cytokine responses after stimulation with a megapool of BP epitopes. Geometric Mean (black line) with geometric standard deviation are shown. Red dotted line represents the threshold or absence of polarization (IL-4/IFNγ ratio of 1). Each dot represents a participant (n=71). B) Graph shows the correlation between the CR and BP Th2 polarization (relative % of IL-4 response). R value and p value are shown by nonparametric Spearman correlation test. The best fit is represented by a linear regression line (red).

**Supplementary Figure 5. Allergen immunodominance is associated with the cytokine profile.** Graphs shows the individual cytokine response directed against the 11 allergens tested, calculated by summing each individual allergen response for IL-4 (A), IFNγ (B), or IL-10 (C). Each bar represents an allergen.

*
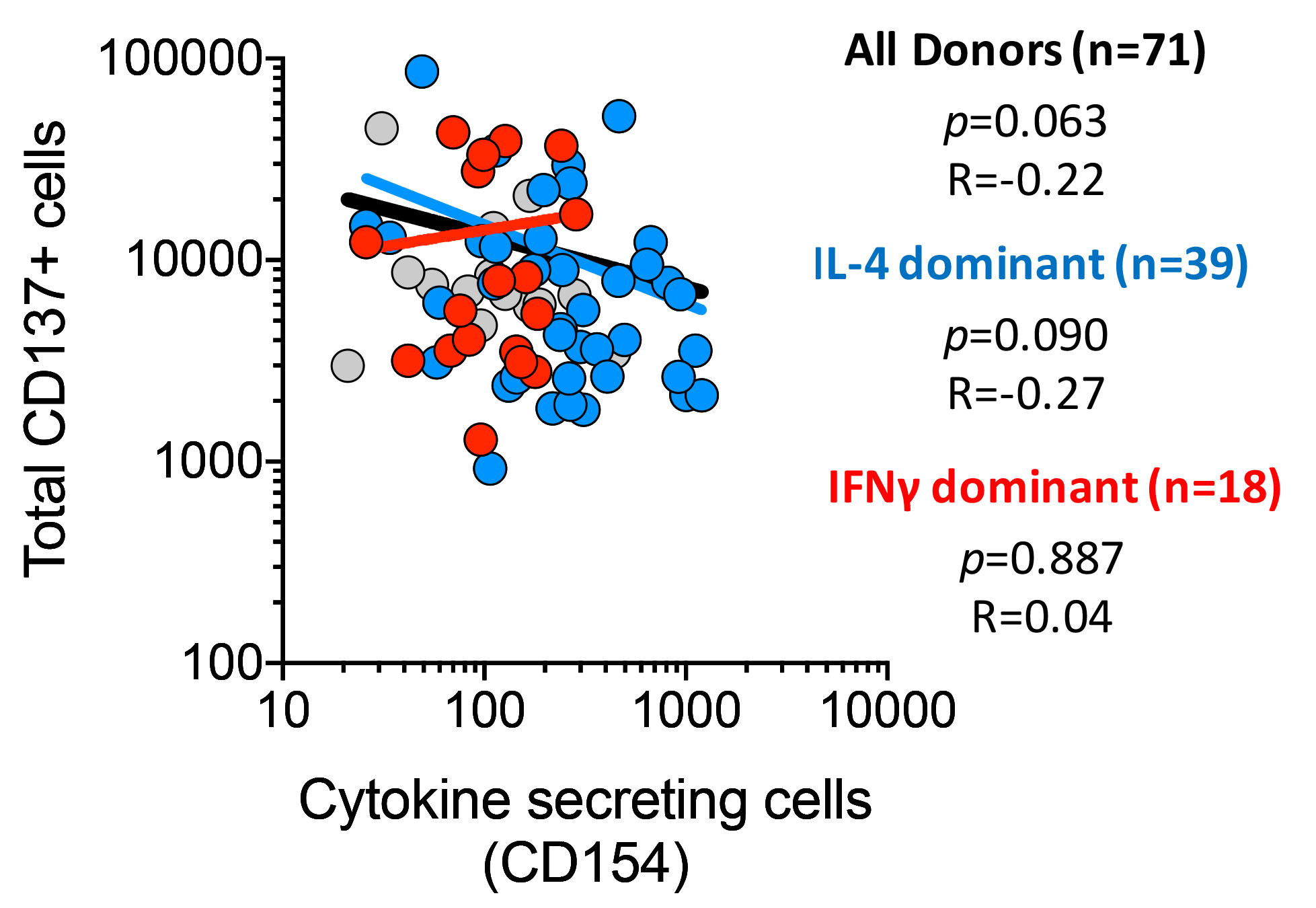
*

**Supplementary Figure 6. Treg numbers are inversely correlated with Teff responses in IL-4 but not IFNγ dominant patients.** Graph shows the correlation of Tregs (Total CD137+ unstimulated cells) and Teff (cytokine+ CD154+ allergen-stimulated) cells by nonparametric Spearman correlation test. Each dot represents a participant (n=71). Blue dots correspond to a subset of donors with IL-4 dominant responses (n=39) and red dots to a subset of donors with IFNγ dominant responses (n=18). R values and p values for all donors or for each subset are shown and best fit represented by a linear regression line.

**Supplementary Figure 7. Clinical symptoms are similar between IL-4 and IFNγ dominant patients.** Graph shows CASI scores (Total or individual components) between the sub-groups of IL-4 (Blue; n=39) or IFNγ (Red; n=18) dominant responders. Each dot represents a participant. Median is shown (black line).

**Supplementary Table 1. Sequences of cockroach allergens used in this study**

Excel file; Supplementary Table 1.xlsx

**Supplementary Table 2. List of Ab used in this study**


**Supplementary Table 3. Correlation of T cell responses with SPT**

**Supplementary Table 4. Correlation of T cell responses with CASI scores**
